# Endoscopic ultrasound-guided antegrade transpapillary multi-hole self-expandable metal stent with fine-gauge stent delivery system

**DOI:** 10.1055/a-2763-5665

**Published:** 2026-01-08

**Authors:** Takeshi Ogura, Jun Matsuno, Takafumi Kanadani, Junichi Nakamura, Hiroki Nishikawa

**Affiliations:** 1 Endoscopy Center, Osaka Medical and Pharmaceutical University Hospital, Osaka, Japan; 2 13010 2nd Department of Internal Medicine, Osaka Medical and Pharmaceutical University, Osaka, Japan

**Fig. 1:**
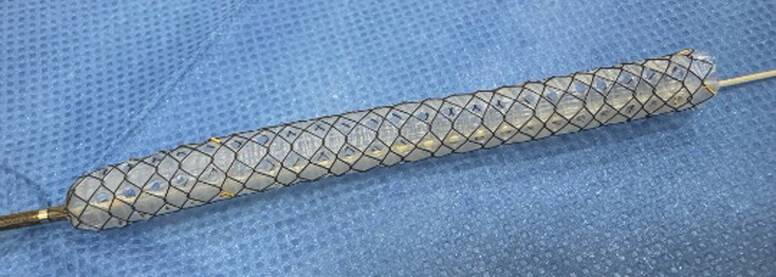
A multi-hole self-expandable metal stent with a 5.9-Fr stent delivery system (MHCSEMS; HANAROSTENT Biliary Multi-hole Benefit; M.I. Tech Co., Ltd, Pyeongtaek, South Korea).

Endoscopic ultrasound-guided antegrade stenting (EUS-AS) can be performed for patients in whom endoscopic retrograde cholangiopancreatography (ERCP) is unsuccessful 1 2 3 . Although EUS-AS has several benefits, including reducing bile leakage from the access route, there are also several concerns. First, stent delivery should be advanced into the biliary tract across the stricture site. Second, if EUS-AS is performed across the papilla, acute pancreatitis should be considered because endoscopic sphincterotomy cannot be performed. Third, to prevent stent dislocation or migration, endoscopic revision may be challenging; therefore, an uncovered metal stent is normally selected, but has limited stent patency. A multi-hole self-expandable metal stent with a fine-gauge stent delivery system (MHCSEMS; HANAROSTENT Biliary Multi-hole Benefit; M.I. Tech Co., Ltd, Pyeongtaek, South Korea) has been developed to overcome these concerns ( Fig. 1 ). The stent is designed to prevent stent migration, through small tissue ingrowths that form in the multiple, small (1.8-mm) side holes along the covering membrane. The side holes also help prevent obstruction of the pancreatic duct. In addition, because the stent delivery system is only 5.9 Fr, it can be advanced easily and smoothly. We describe herein technical tips for EUS-AS using the MHCSEMS.

**Fig. 2:**
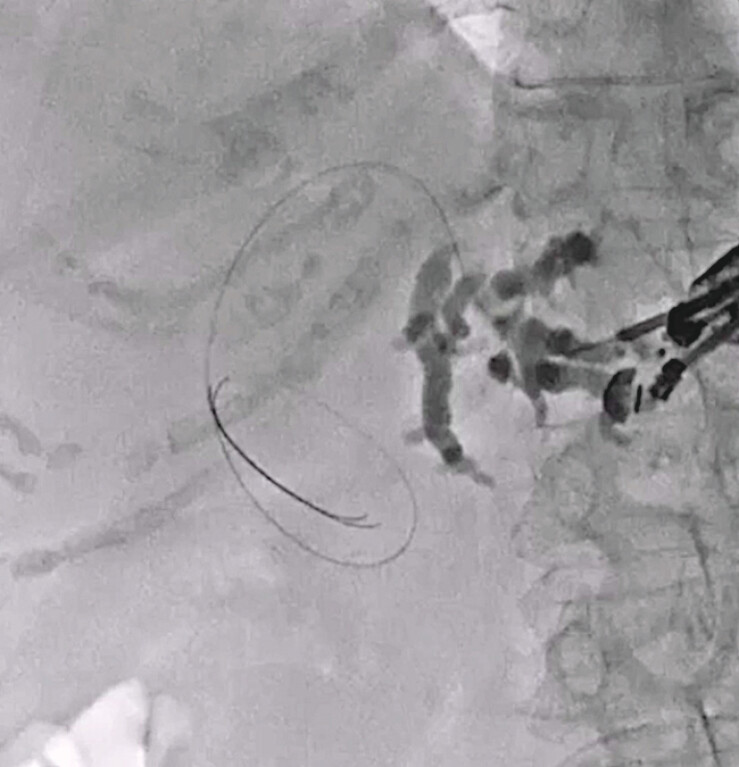
A guidewire is deployed under endoscopic ultrasound guidance.

**Fig. 3:**
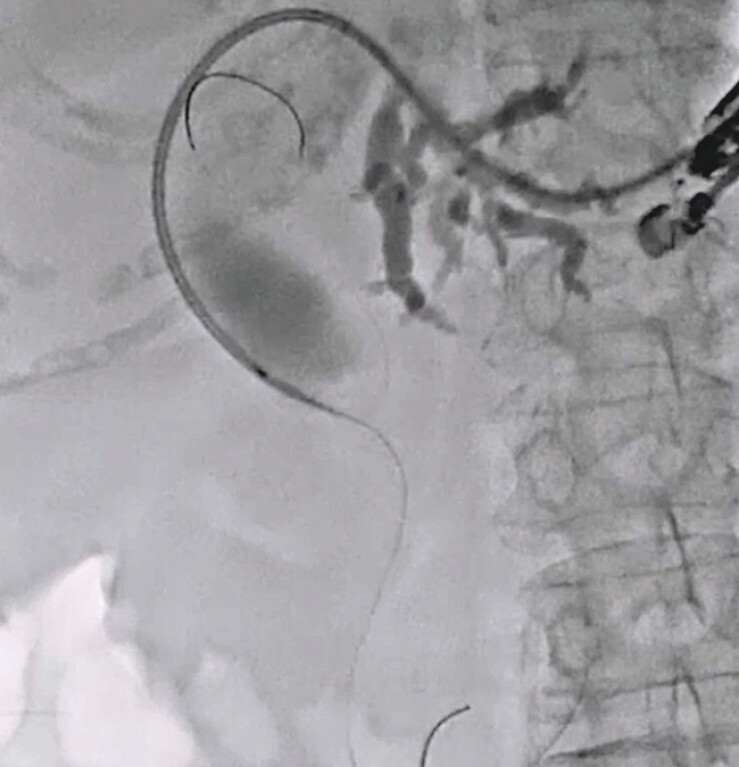
A double lumen dilator is inserted into the biliary tract.

**Fig. 4:**
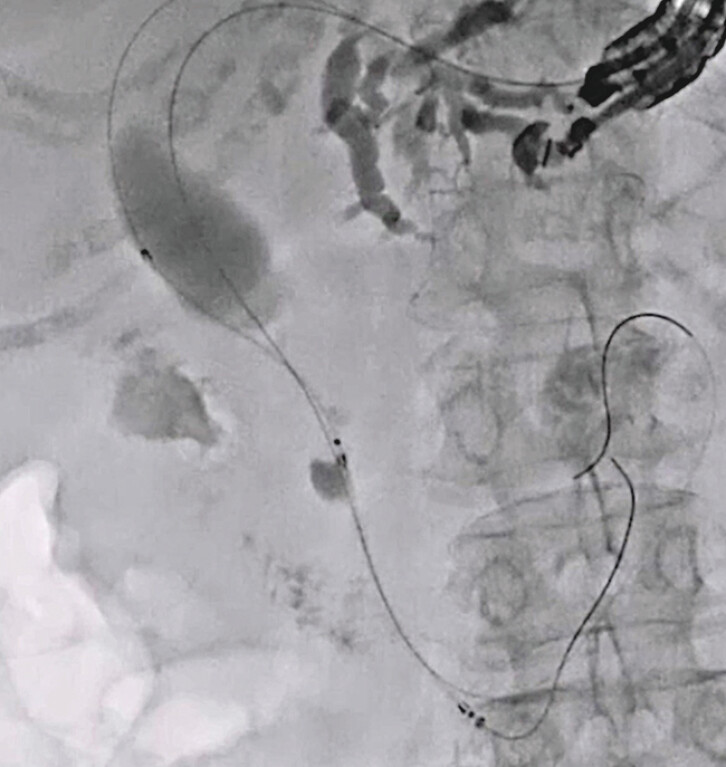
A stent delivery system is inserted across the papilla.

**Fig. 5:**
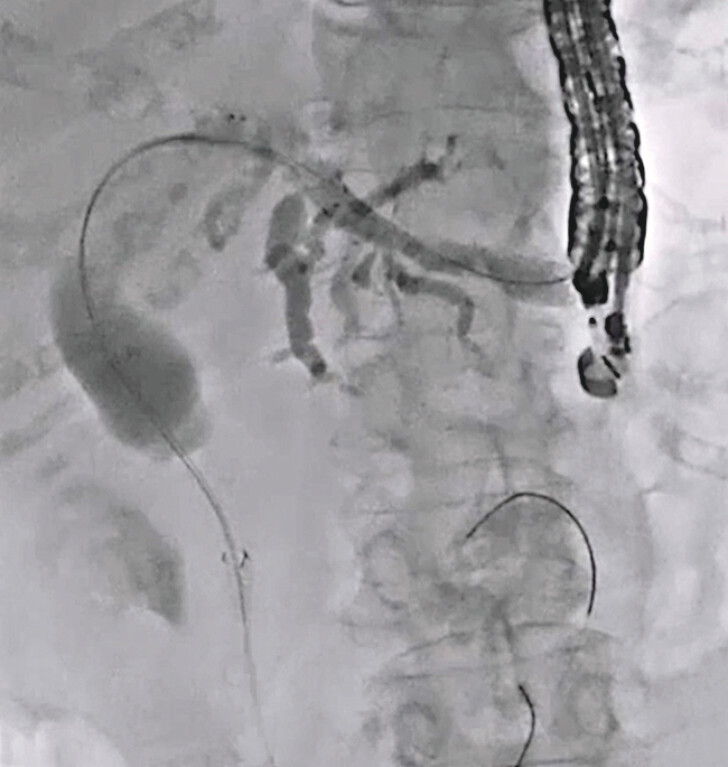
Endoscopic ultrasound-guided hepaticogastrostomy is performed.

The dilated intrahepatic bile duct was punctured using a 19G needle, and a 0.025-inch guidewire was inserted. A double lumen dilator was then inserted ( Fig. 2 ). Cholangiography identified lower bile duct obstruction as a complication. After performing the double guidewire technique, we attempted to advance the guidewire into the intestine across the papilla ( Fig. 3 ). The MHCSEMS stent delivery was then inserted easily ( Fig. 4 ). After successful EUS-AS, EUS-guided hepaticogastrostomy was performed without any adverse events ( Fig. 5 ; Video 1 ). No acute pancreatitis or stent dislocation was observed during follow-up.

Antegrade transpapillary multi-hole self-expandable metal stent with fine-gauge stent delivery system is performed.Video 1

In conclusion, EUS-AS using the MHCSEMS with a fine-gauge stent delivery system appears to be a suitable insertion technique that prevents pancreatic duct obstruction and stent dislocation.

Endoscopy_UCTN_Code_TTT_1AS_2AH
